# Association of sleep bruxism with obstructive sleep apnea and gastroesophageal reflux disease: a cross-sectional study in Egyptian governmental dental clinics

**DOI:** 10.1186/s12903-026-07847-0

**Published:** 2026-02-26

**Authors:** Hadiel Zamzam, Eman M. Ibreheem, Asmaa N. Elboraey, Mennatallah Mohamed Elhoteiby, Ahmed Gharib, Enas Sabry, Noha Adel, Ali Yasser, Magda I. Ramzy, Amani R. Moussa

**Affiliations:** 1 Department of Fixed and Removable Prosthodontics, Oral and Dental Research Institute, National Research Centre, Cairo, Egypt; 2 Department of Internal Medicine, Medical Research and Clinical Studies Institute, National Research Centre, Cairo, Egypt; 3 Faculty of Dentistry, Ain-Shams University, Cairo, Egypt; 4 Faculty of Oral and Dental Medicine, Cairo University, Cairo, Egypt; 5 Faculty of Dentistry, Suez University, Suez, Egypt

**Keywords:** Sleep bruxism, OSA, GERD, Sleep disorders, Egypt

## Abstract

**Objectives:**

The objective of this cross-sectional study was to quantify the prevalence of sleep bruxism (SB) in an Egyptian population sample and to identify possible associations between SB, obstructive sleep apnea (OSA), and gastroesophageal reflux disease (GERD).

**Methods:**

This study was conducted on patients visiting dental clinics of three governmental institutions: the National Research Centre, Cairo University, and Ain-Shams University in Egypt. All dentulous patients aged 18–70 were included in the study; those with physical or mental disabilities or genetic disorders were excluded. Validated research-made questionnaires and clinical dental examinations were used to diagnose SB, OSA, and GERD. Statistical analysis was performed to study possible associations. The significance level was set at *p* ≤ 0.05.

**Results:**

Nine hundred ninety-one subjects participated in this study. The prevalence of SB was 13.0%. The mean and standard deviation (SD) of the OSA score in sleep bruxers were 1.48 (1.55), as opposed to 1.35 (1.5) for non-sleep bruxers, showing no statistical significance with *p* = 0.388. The prevalence of GERD in the population was 22.7%; in sleep bruxers, 36.4% and in non-sleep bruxers, 20.7%, showing high statistical significance. The mean GERD score for sleep bruxers was 7.24 (2.36), as opposed to 6.69 (1.79) for non-sleep bruxers, also showing statistical significance with *p* = 0.002.

**Conclusions:**

The Egyptian population has a high prevalence of SB (13.0%). An association between SB and GERD was manifested. However, no relation between SB and OSA was found.

**Supplementary Information:**

The online version contains supplementary material available at 10.1186/s12903-026-07847-0.

## Introduction

Bruxism is a repetitive jaw-muscle activity characterized by clenching or grinding teeth and bracing or thrusting the mandible [1]. It is considered to have a multifactorial etiology that includes poorly defined aspects of central nervous system function and genetic and behavioral factors. There is currently no proven treatment that can effectively and permanently end bruxism. Current treatment focuses on symptom management and prevention of further complications. The most used management approaches are intraoral devices [2, 3], physical therapy [4], botulinum toxins injections [5–7], pharmacotherapy [8–10], and behavioral strategies [11]. 

Bruxism is considered among the most detrimental parafunctional activities of the stomatognathic system, as it is responsible for tooth wear, periodontal tissue lesions, and muscular damage. These manifestations, together with fractured teeth, headaches, and TMJ disturbances, affect the general health and, consequently, the quality of life [12]. Severe bruxism is often associated with mechanical and technical complications in cosmetic dentistry and prosthetic rehabilitation. Patients who present with severe tooth wear often have severe aesthetic issues and loss of occlusal vertical dimension, which may necessitate multidisciplinary rehabilitation strategies. Hence, early diagnosis and intervention are strongly recommended to prevent or delay further disease development.

Two types of bruxism exist sleep and awake bruxism. There is evidence that sleep bruxism (SB) is associated with other sleep disorders, such as obstructive sleep apnea (OSA) [13] and Gastroesophageal reflux disease (GERD) [14]. Obstructive sleep apnea is a respiratory tract syndrome characterized by repetitive upper airway obstruction, followed by hypoxia and various degrees of arousal. Snoring, choking, and gasping for air are some of the signs and symptoms of OSA. Moreover, recurrent awakenings and insomnia lead to daytime sleepiness, fatigue, and concentration impairment. Treatment of mild OSA includes mandibular advancement devices (MAD), nasal resistors, positional therapy, and behavioral changes. Severe cases of OSA are managed by continuous positive airway pressure (CPAP) or are treated surgically [15]. 

Epidemiological research shows that around 20% of the general population experiences GERD once a week [16, 17]. GERD has considerable consequences on hard tooth structure in the form of chemical wear (i.e., erosion) and subsequent tooth hypersensitivity [18]. It can also exacerbate xerostomia and hypersalivation, with choking being a possible consequence of the latter [19]. GERD is, furthermore, associated with causing or aggravating snoring and OSA, although evidence of a causal connection has yet to be found [20]. 

The association between sleep disorders has been extensively investigated in the literature; [21–28] however, the precise pathophysiological and temporal relationships remain incompletely understood and are yet to be fully elucidated. For example, SB may serve a protective function in OSA by activating the masseter muscles to reposition the jaw and increase upper airway patency, thereby potentially interrupting respiratory events. Additionally, GERD appears to exacerbate OSA severity, implying a bidirectional influence among these disorders. Due to their frequent co-occurrence and clinical interplay, these conditions pose important considerations for dental practice, where they are seldom encountered in isolation and require an interdisciplinary approach for effective management [29]. 

Although bruxism is generally a common condition with a reported high prevalence, there is a significant lack of epidemiological data about SB and other sleep disorders in Egypt. Thus, this study was conducted to quantify SB’s prevalence and investigate its association with other sleep disorders in a sample of the Egyptian population. A survey composed of validated research-made questionnaires and dental examination were used to identify the three conditions: SB, OSA and GERD. The null hypothesis stated that SB, OSA and GERD are independent of one another in a sample of the Egyptian population.

## Materials and methods

### Study settings and locations

This study was part of a research project (ID number: 12060208) studying oral health in a sample of the Egyptian population regarding sleep disorders. The National Research Centre in Egypt (NRC) funded the project, which agreed with the Code of Ethics of the World Medical Association, following the guidelines and regulations of Helsinki Declaration 1975. The study was approved by the NRC Medical Research Ethics Committee (MREC ethical approval number: 19242). All patients were informed with the current study’s practical phases and signed a printed consent of their approval.

This cross-sectional study was conducted on patients visiting dental clinics of three governmental institutions in Egypt: The National Research Centre, Cairo University, and Ain-Shams University. The study consisted of a survey and dental examination. The survey comprised of clinician-administered validated research-made questionnaires for identifying SB, OSA, and GERD.

### Eligibility criteria for participants

All participants signed an informed consent form for their approval. The data were collected from January 2020 to April 2022. All dentulous patients between the ages of 18 and 70 visiting the previously stated clinics were asked to participate in the study. Individuals with physical or mental disabilities, as well as those with genetic disorders, were excluded from the study.

### Pilot study

A pilot survey was first implemented to assess the questions’ clarity, relevance, and sequence. Questions were translated into Arabic, adapting to the patient’s language and knowledge. Modifications were made based on feedback from the examiners and participants. A standardized team of eight examiners was trained on the examination method to ensure the reliability of the data.

### Sample size calculation

According to the results of a previous study [30], in which the prevalence of bruxism was reported to be 31.4%, and by adopting a confidence interval of 97% and a margin of error of 3.5% with finite population correction, the predicted sample size (n) was 827 subjects. The total sample size increased to 952 to compensate for a 15% dropout. The software Epi INFO version 7.2.5.0 (CDC, Georgia, USA) was used for this calculation.

### Demographic data

The demographic data regarding age, sex, and occupation were collected from all participants.

### Bruxism

The diagnosis of SB was conducted based on the diagnostic criteria of the American Academy of Sleep Medicine [31]. SB was diagnosed by (1) a self-reported questionnaire and (2) a dental clinical examination. The questionnaire yielded yes/no responses to the following questions, as shown in Table 1.

**Table 1:** Bruxism questionnaire

Questions	Yes	No
Are you aware of grinding/clenching your teeth frequently during sleep?		
Has your partner told you that you make grinding noises during sleep?		
Do you have any of the following symptoms upon awakening: • sensation of fatigue, tightness, or soreness of your jaw • feeling that your teeth are clenched or that your mouth is sore • aching of your temples • difficulty in opening your mouth wide • feeling of tension in your jaw joint and feeling as if you have to stretch your lower jaw to release the tension • hearing or feeling a ‘click’ in your jaw joint that may disappear afterward?		

The dental clinical examination assessed the following signs of bruxism: abnormal tooth wear, impressions of teeth in the buccal region, impressions of teeth on the lateral borders of the tongue, and masseter muscle hypertrophy.

The presence of SB was defined when at least one of the two first questions, about the awareness of grinding teeth at night, was answered by yes or when it was answered by no, but there were two or more positive answers in the third question and clinical examination, indicating the presence of clear signs and symptoms of bruxism.

### Obstructive Sleep Apnea (OSA)

The risk of OSA was assessed using the STOP-BANG questionnaire [32]. Participants answered yes/no questions about:

- *S*noring.
- *T*iredness during daytime.
- *O*bservation of stopped breathing during sleep.
- high blood *P*ressure.
- *B*ody mass index of more than 35 kg/m^2^.
- *A*ge more than 50 years old.
- *N*eck circumference larger than 40 cm.
- male *G*ender.

According to the OSA scoring system by the STOP-BANG questionnaire, a participant was considered at:

• low risk of OSA when 0–2 answers were yes;
• intermediate risk of OSA when 3–4 answers were yes;
• high risk of OSA when 5–8 answers were yes;
  - or two or more answers to the STOP questions were yes, supported with male gender, BMI > 35 kg/m2, or neck circumference > 40 cm.

### Gastro-Esophageal Reflux Disease (GERD)

The GERDQ questionnaire [33] was used to assess the GERD score of individuals. It consists of six questions about the incidence of GERD symptoms: heartburn, regurgitation, epigastric pain, nausea, sleep disturbance, and use of over-the-counter medications within the previous seven days. Each answer took a score, as shown in Table 2. The total sum of the patient’s scores showed their likelihood of having GERD. A total sum of 0–2 points describes a 0% likelihood of GERD; 3–7 points describe a 50% likelihood; 8–10 points represent 79% likelihood; and 11–18 points represent 89% likelihood of GERD. Hence, 8 points or above describe the possibility of having GERD.

**Table 2:** GERDQ questionnaire and scoring system

**Questions**	**Symptom presence**			
	**0** **days**	**1** **day**	**2–3** **days**	**4–7** **days**
1. How often did you have a burning feeling behind your breastbone?	0	1	2	3
2. How often did you have stomach contents moving upwards to your throat or mouth?	0	1	2	3
3. How often did you have pain in the center of the upper stomach?	3	2	1	0
4. How often did you have nausea?	3	2	1	0
5. How often did you have difficulty sleeping well because of your heartburn and/or regurgitation?	0	1	2	3
6. How often did you take additional medication for your heartburn and regurgitation other than what the physician told you to take (such as Maalox)?	0	1	2	3

### Statistical analysis

The statistical analysis aimed to compare the risk of OSA and the likelihood of GERD in sleep bruxers versus non-sleep bruxers and to analyze associations between the three sleep disorders. Statistical analysis was performed with IBM SPSS Statistics for Windows, Version 23.0. Armonk, NY: IBM Corp. Qualitative data were presented as frequencies and percentages. The Chi-square test was used to study different associations. Numerical data were presented as median, mean, and standard deviation (SD) values. The students’ t-test was used to compare the two groups. The significance level was set at *p* ≤ 0.05.

The ROC (Receiver Operating Characteristic) curve was constructed to determine the diagnostic accuracy of questions about bruxism symptoms upon awakening. ROC curve analysis was performed with MedCalc^®^ Statistical Software version 19.5.1 (MedCalc Software Ltd, Ostend, Belgium; https://www.medcalc.org; 2020).

## Results

The present study was conducted on 991 subjects: 391 males (39.5%) and 600 females (60.5%). The mean and standard deviation (SD) values for age were 37.8 (11.5) years old, with a minimum of 18 and a maximum of 70 years old. The most frequent occupation was white-collar jobs (41.8%), followed by unemployed participants (32.2%), while 1% did not report their jobs.

**Fig. 1:**
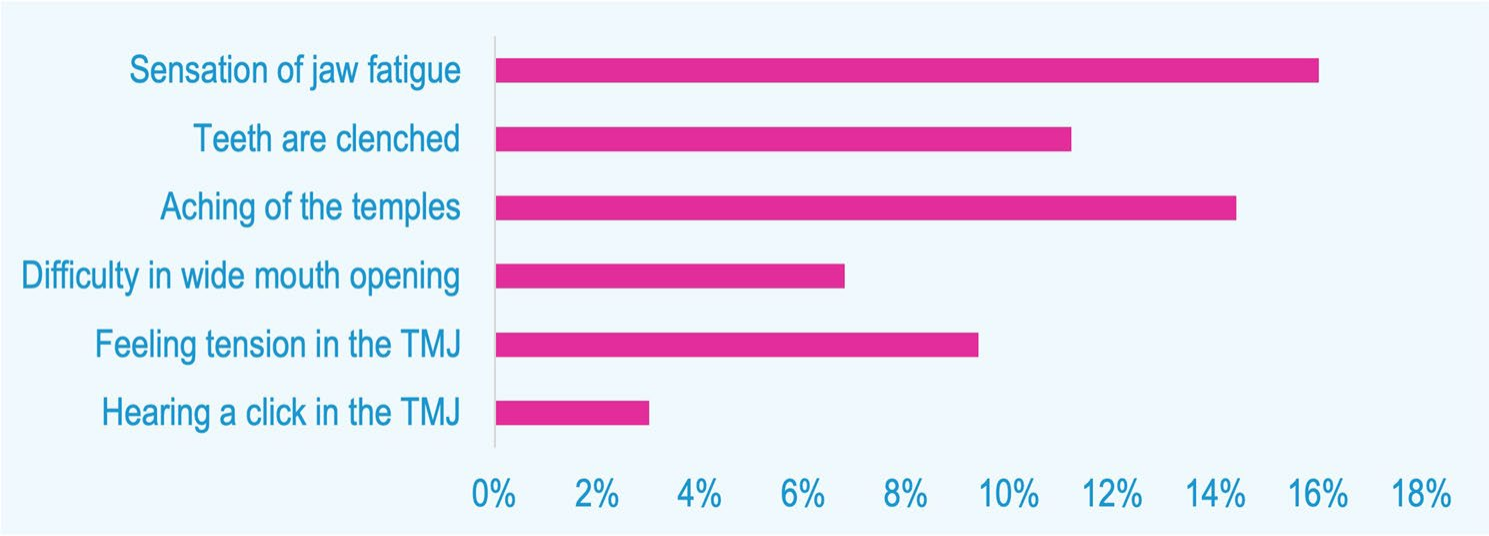
Prevalence of symptoms of SB upon awakening in the surveyed population

The prevalence of SB in the population sample was 13.1%. Questions related to the symptoms of SB upon awakening had the following responses (Fig. 1).

The average age of sleep bruxers was 38.0, as opposed to 37.7 for non-sleep bruxers. There was no statistically significant difference between mean age values in patients with and without SB (*p* = 0.78). Concerning gender, there were significantly more females (73.1%) among sleep bruxers than males (26.9%). A smaller difference exists in non-sleep bruxers, showing a statistically significant association of SB with gender (*p* = 0.002) (Table 3).

**Table 3:** Descriptive statistics and results of student’s t-test for comparison between gender and age values in subjects with and without SB

	SB (*n* = 129)		Non-SB (*n* = 860)		*p*-value
Age	mean	SD	mean	SD	0.78
	38.0	10.4	37.7	11.7	
Gender (in percentage)	males	females	males	females	0.002*
	26.9%	73.1%	41.3%	58.7%	

*: Significant at *p* ≤ 0.05

### OSA

**Fig. 2:**
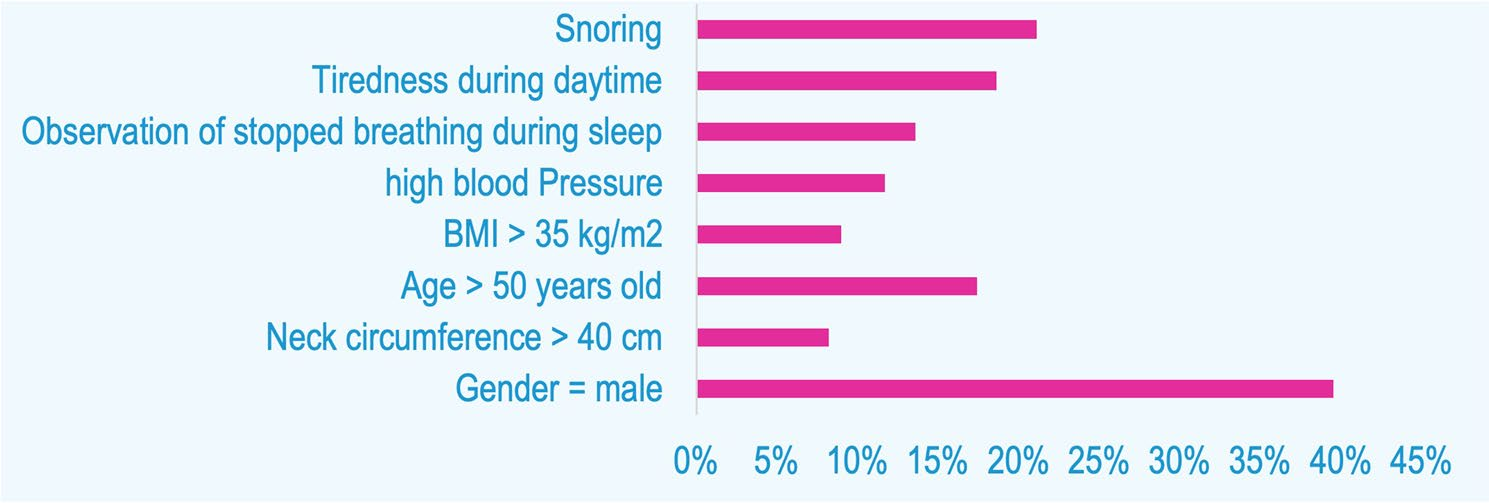
Descriptive statistics for sleep apnea (*n* = 991)

Results of the STOP-BANG questionnaire and descriptive statistics for sleep apnea are as follows (Fig. 2).

The mean of OSA score in sleep bruxers was 1.48 (SD = 1.55), as opposed to 1.35 (SD = 1.5) for non-sleep bruxers. The Student’s t-test, used to compare OSA scores in participants with and without SB, revealed no statistically significant association between SB and OSA with *p* = 0.388. In subjects with SB, low risk of sleep apnea was the most prevalent degree, followed by intermediate, then high risk. However, this is only a clinical observation. The Chi-square test resulted in *p* = 0.905, indicating no statistical significance within the groups (Table 4).

**Table 4:** Descriptive statistics and results for comparison between OSA in participants with and without SB

Sleep apnea	SB (*n* = 129)		Non-SB (*n* = 860)		*p*-value	SB (*n* = 129)	Non-SB (*n* = 860)		*p*-value
	*n*	%	*n*	%		OSA score mean (SD)	OSA score mean (SD)		
Low risk	104	80.8	707	82.2	0.905	1.48 (1.55)	1.35 (1.50)		
Intermediate risk	17	13.1	107	12.4					0.338
High risk	8	6.2	46	5.3					

*: Significant at *p* ≤ 0.05

### GERD

The mean and SD values of the GERDQ score were 6.76 (SD = 1.88) with a median of 6 and a range between 1 and 18. The prevalence of GERD in the population (scores equal to or higher than 8) was 22.7%, 4.7% of which were SB. Participants with SB showed a higher prevalence of 0%, 79%, and 89% likelihood of GERD than other subjects. The mean GERDQ score for sleep bruxers was 7.24 (2.36), as opposed to 6.69 (1.79) for non-sleep bruxers, showing a statistically significant association with *p* = 0.002 via the Student’s t-test. Among sleep bruxers, 36.4% had GERD, while among non-sleep bruxers, only 20.7% had GERD. Chi-square showed a statistically significant association between SB and the likelihood of GERD with *p* < 0.001 (Table 5).

**Table 5:** Descriptive statistics and results showing the association between SB and the likelihood of GERD

Likelihood of GERD	SB (*n* = 129)		Non-SB (*n* = 860)		*p*-value	SB (*n* = 129)	Non-SB (*n* = 860)		*p*-value
	*n*	%	*n*	%		GERDQ score mean (SD)	GERDQ score mean (SD)		
0%	4	3.1	3	0.3	< 0.001*	7.24 (2.36)	6.69 (1.79)		0.002*
50%	78	60.5	679	79					
79%	32	24.8	138	16					
89%	15	11.6	40	4.7					

*: Significant at *P* ≤ 0.05

### Diagnostic accuracy of the six bruxism symptoms questions

**Fig. 3:**
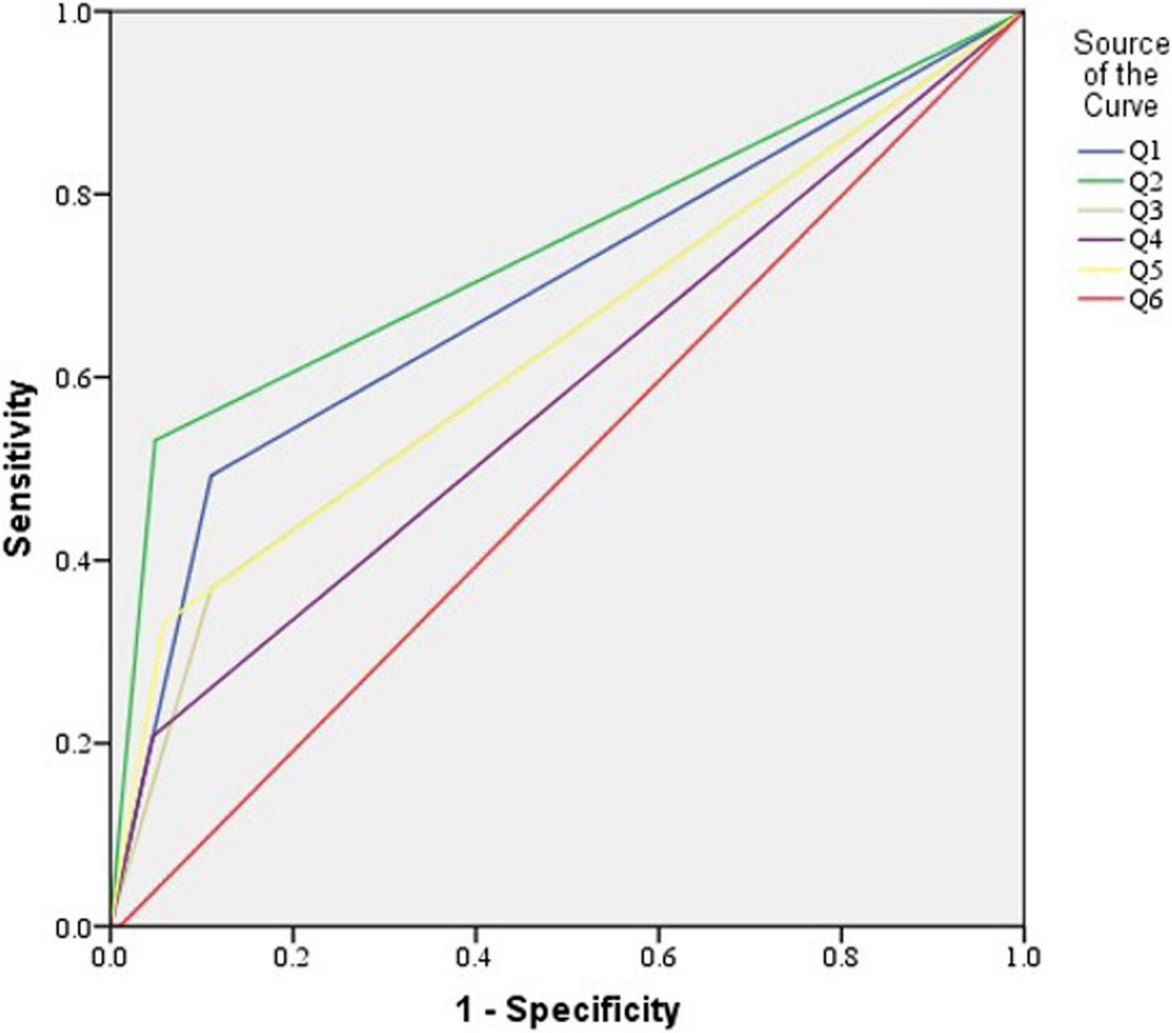
ROC curve of the six questions of bruxism symptoms upon awakening: (1) sensation of fatigue, tightness, or soreness of your jaw, (2) feeling that your teeth are clenched or that your mouth is sore, (3) aching of your temples, (4) difficulty in opening your mouth wide, (5) feeling tension in your jaw joint and feeling as if you have to move your lower jaw to release it; (6) hearing or feeling a ‘click’ in your jaw joint that disappears afterward

ROC curve analysis of the six questions about bruxism symptoms upon awakening is presented in Table 6; Fig. 3. ROC curve analysis showed that the highest diagnostic accuracy was obtained with Question 2 (89.6%), followed by Question 5 (86.2%), while the least accuracy was obtained with Question 3 (82.2%).

**Table 6:** Sensitivity, specificity, predictive values, diagnostic accuracy, area under the ROC curve (AUC) and 95% confidence interval (95% CI) of the (AUC) for the six questions of bruxism symptoms upon awakening: (1) sensation of fatigue, tightness or soreness of your jaw, (2) feeling that your teeth are clenched or that your mouth is sore, (3) aching of your temples, (4) difficulty in opening your mouth wide, (5) feeling tension in your jaw joint and feeling as if you have to move your lower jaw to release it; (6) hearing or feeling a ‘click’ in your jaw joint that disappears afterward

Question	Sensitivity %	Specificity %	+PV %	-PV %	Diagnostic accuracy %	AUC	95% CI
Question 1	49.2	89.0	40.3	92.1	83.8	0.691	0.661–0.72
Question 2	53.1	95.1	62.2	93.1	89.6	0.741	0.713–0.768
Question 3	36.9	89.0	33.6	90.3	82.2	0.629	0.599–0.66
Question 4	20.8	95.4	40.3	88.9	85.6	0.581	0.549–0.612
Question 5	33.1	94.2	46.2	90.3	86.2	0.636	0.606–0.666
Question 6	2.3	96.6	10.0	86.8	84.3	0.504	0.473–0.536

*+PV* Positive Predictive Value, *-PV* Negative Predictive Value

## Discussion

In 2018, an international consensus on the assessment of bruxism was published, stressing that bruxism should not be considered a disorder anymore [34]. In 2021, Manfredini et al. [35] stated that the bruxism construct shifted the focus from bruxism being a parafunction/disorder to being a muscular activity that can be a risk and a protective factor for certain clinical consequences. With this new conceptualization of bruxism, the need for more cross-sectional and longitudinal studies to understand more about bruxism and its association with specific clinical conditions cannot be neglected. For this purpose, this study was designed to first quantify the prevalence of SB in a sample of the Egyptian population and to identify the interrelations of sleep bruxism with other sleep disorders, specifically OSA and GERD.

Different methods can carry out the diagnosis of bruxism. It is accepted that polysomnography is the gold standard for diagnosing definite SB [36]. However, its cost and inaccessibility limit its use to small cohorts. A practical and applicable tool for screening sleep and awake bruxism is self-reported research-made questionnaires. In 2022, a standardized tool for assessing bruxism (STAB) [36] was proposed to evaluate the status of bruxism, comorbid conditions, etiology, and consequences. It includes a subject-based assessment based on patients’ self-report, a clinically based evaluation by an examiner, and an instrumentally based assessment through sleep-time electromyography (EMG), polysomnography (PSG), or both [37]. In this cross-sectional study, self-reported research-made questionnaires, as well as clinical examinations, were performed. The instrumentally based assessment was excluded at this time; however, it will be included in future research. The questionnaire for the diagnosis of SB was composed of two yes/no questions and awakening symptoms questions suggested by the American Academy of Sleep Medicine [31] about six possible symptoms of SB. The awakening symptoms questions and the clinical examination were responsible for determining the SB status of participants, who and whose bedpartners are unaware of tooth grinding habits.

Although the awakening symptoms questions have been recommended by the American Academy of Sleep Medicine [31], no information on the interpretation of answers has ever been reported. Hence, the ROC analysis in this study is the first step in evaluating the accuracy of the awakening symptoms questions for diagnosing SB. According to the results, all six questions had an accuracy greater than 80%. The question with the highest accuracy was about the feeling that the teeth are clenched, most probably because it is a noticeable feeling. All six questions had relatively high specificity. While ROC analysis is useful for evaluating the internal consistency or discriminative ability of the questions, in the absence of polysomnography as the gold standard, it cannot serve as a substitute for true diagnostic validation. Additional research is recommended to validate the scoring system for SB.

Due to demographic reasons and different methods of diagnosis, a wide range of the prevalence of bruxism can be found in the literature. In 2000, Bader et al. [38] stated that 85–90% of the population experiences tooth clenching or grinding at some point in their lifetime. Frequent grinding was only found in 8% of adults in the population, according to Lavigne et al. [39], Maluly et al. [40]. , and Carra et al. [41]. Other studies reported a higher prevalence of SB. According to a systematic review by Manfredini et al. [42], the prevalence of sleep bruxism ranged from 8% to 31.4% in two studies and averaged 12.8% in three other studies. In comparison, the prevalence of awake bruxism ranged from 22.1% to 31% in two studies. According to this study, the 13.1% prevalence of SB in a sample of the Egyptian population coincides with the three studies mentioned in the systematic review [42]. The method of bruxism diagnosis could be held accountable for the homogeneity of data. The previously mentioned studies, including this one, were based on self-reported data using research-made questionnaires. When polysomnography was added to confirm the diagnosis, the numbers drastically decreased [40]. Although polysomnography might be a more objective tool for the diagnosis of bruxism, its exclusion with larger sample sizes is sometimes necessary.

Regarding age, this study showed no positive association between SB and the average age of participants. This finding coincides with the results of Bayer et al. [43], also reporting an insignificant association between age and bruxism. Unlike other reports in the literature [39, 44, 45] that show no gender difference in the prevalence of SB, this study had a markedly higher female percentage of sleep bruxers. Similarly, a recent study [46] about the relationship between emotional stress and sleep/ awake bruxism among dental students revealed a higher SB prevalence in females than in males and, conversely, a higher awake bruxism prevalence in males. Given the potentially different etiologies of awake and sleep bruxism, more screening tests are needed for gender comparison that deal with awake and sleep bruxism as two different conditions.

### OSA

The global prevalence of sleep apnea is 48%, according to an analysis conducted by Benjafield et al. in 2019 [47]. The gold standard for its diagnosis is polysomnography, which can differentiate between mild, moderate, and severe sleep apnea depending on the number of events per hour and oxygen desaturation. The problems related to the high cost, the equipment required, and the overnight stay of sleep studies pushed for the evolution of other more straightforward diagnostic tools. The STOP-BANG questionnaire, for example, is a validated test for assessing the risk of individuals for OSA. Its scoring system assigns individuals into categories of low, intermediate, and high risk of OSA. The causal relationship between OSA and SB is still unclear. It was recently stated that SB could be a risk factor or a protective mechanism for OSA [34]. The most common hypothesis is that hypoxia related to OSA triggers rhythmic masticatory muscle activity (RMMA) in an attempt from the body to clear the airway. Scientific evidence is still lacking [48]. 

The method of identification of OSA, the STOP-BANG questionnaire, did not allow us to quantify its prevalence in the Egyptian population. And it was noticed that few participants could identify with sleep apnea symptoms. This could be attributed to the poor awareness of sleep disorders, especially among socially and educationally less fortunate individuals in Egypt. Additionally, the sample selected was patients seeking dental check-ups and treatment, not necessarily complaining of any respiratory conditions. A closer inspection of the randomly collected sample in this study reveals a female and a younger age dominance, which constitutes one of the study’s limitations, given that OSA has a higher incidence in males and old age. These clinical findings are reflected in the population’s high percentage of low-risk OSA. Other limitations of this study are related to self-reported data, sample selection, the lack of polysomnographic confirmation of OSA and the presence of potential confounding factors, such as age, gender, BMI and smoking. Hence, further studies that use a more objective diagnostic method and, more specifically, screened samples to quantify and analyze the disorder are highly recommended.

The surveyed sample reported a high incidence of snoring, which is sometimes considered an early phase of OSA. Although snoring was found to increase the probability and severity of OSA, controversial reports in the literature about a direct effect on SB exist [48]. 

Recently, the relationship between SB and OSA has been thoroughly studied. Some studies confirm a direct association between them [49–51]. Wojoda et al. [52] and Hosoya et al. [53] demonstrated a positive correlation between the incidence of OSA and the episodes of SB diagnosed by polysomnography. Jokubauskas et al. [54] also contended that there is a relationship between SB and sleep apnea but did not confirm it due to a lack of scientific evidence. For the same reason, Da Costa Lopes et al. [55] did not establish any link between SB and sleep apnea, which agrees with our results. Within the limitations of our study, its results showed no statistically significant association between SB and OSA.

In 2022, Kazubowska-Machnowska et al. [56] studied the effect of OSA on SB and found that their relationship depended on the severity of OSA. SB was observed in mild and moderate OSA. To investigate the possible relationship of SB with the degree of severity of OSA in our sample population, the number of SB and non-sleep bruxers were compared within the groups of low, moderate, and high risk of OSA. Unlike the earlier paper, our comparisons revealed no statistically significant relationship between SB and OSA with its different categories. It should be underscored that the screening method used here is different from polysomnography, that can accurately identify and classify a patient with OSA. Instead, the STOP-BANG questionnaire can only record the risk of OSA, which is one of the limitations of this study and a possible reason for the statistically non-significant results related to OSA. Hence, data should be carefully interpreted.

### GERD

A validated questionnaire commonly used to diagnose GERD was used in this study. The GERDQ has a sensitivity of 65% and a specificity of 71% for GERD diagnosis [57]. The prevalence of GERD in the studied sample was 22.7%. This is consistent with a comprehensive review reporting that GERD affects between 8.7 and 33.1% of the Middle Eastern population [58]. 

There is strong evidence that GERD and SB are related to one another, with GERD events preceding bruxism events [14, 59]. Previous studies showed that when the esophageal pH value is reduced below 5.0, the number of RMMA episodes increases [14, 59]. The masticatory muscle activity is thought to help stimulate the secretion of saliva and the resulting increase in the pH value. On this basis, it has been suggested that SB results from acidic reflux, arising through arousal, often together with swallowing. In agreement with other articles [14, 60–62], this cross-sectional study shows a strong association between SB and GERD. The incidence of GERD was significantly higher in sleep bruxers than in non-sleep bruxers. This study cannot explain this association. However, it proves that the two conditions share a common pathophysiological pathway or a contributing factor [63]. 

## Conclusions

This is the first study to survey SB in the Egyptian population. Within the limitations of this study, 13% of the studied population were diagnosed as sleep bruxers. The awakening symptoms questions recommended by the American Academy of Sleep Medicine showed a high accuracy for diagnosing SB. A relatively low risk of OSA was found in the surveyed Egyptian population, and there was no positive association between OSA and SB. Conversely, a high prevalence of GERD was observed in the studied sample with a strong association with SB. Further research is needed to investigate the physiological link between SB and GERD, particularly through longitudinal studies to establish causality.

This study highlights the important role of dental clinicians and gastroenterologists in early identification of sleep disorders, which could facilitate multidisciplinary interventions to mitigate compounded risks.

## Supplementary Information

Supplementary Material 1.

## Data Availability

Availability of data and materials of the datasets used and analyzed during the current study are available from the corresponding author upon reasonable request.
