# Supplementary material for: Association of sleep bruxism with obstructive sleep apnea and gastroesophageal reflux disease: a cross-sectional study in Egyptian governmental dental clinics
Source: BMC Oral Health. 2026 Feb 26;26:546. doi: 10.1186/s12903-026-07847-0 (PMC13033221; doi:10.1186/s12903-026-07847-0)
Supplement: Supplementary file 1 — Supplementary Material 1. [file 12903_2026_7847_MOESM1_ESM.docx]

**Sleep bruxism and its association with obstructive sleep apnea and gastroesophageal reflux disease: a cross-sectional study in Egyptian governmental dental clinics**

Asmaa N. Elboraey^1^, Amani R. Moussa,^1^ Eman Mostafa,^1^Menna Hoteiby^1^, Ahmed Gharib^2^, Enas Sabry^3^, Noha Adel^4^, Ali Yasser^4^, Magda I. Ramzy,^1^ Hadiel Zamzam^1^

**Research article**

**Authors:**

1-Department of Prosthodontics, Oral and Dental Research Institute, National Research Centre, Cairo, Egypt.

2-Department of Internal Medicine, Medical Research and Clinical Studies Institute, National Research Centre, Cairo Egypt.

3- Faculty of Dentistry, Ain-Shams University, Cairo Egypt.

4-Faculty of Oral and Dental Medicine, Cairo University, Cairo Egypt.

**Corresponding author: Asmaa N. Elboraey**

E-mail: [asmaanabil10@yahoo.com](mailto:asmaanabil10@yahoo.com)

Tel: +02-33371635

**ABSTRACT**

**Objectives:**

The objective of this cross-sectional study was to quantify the prevalence of sleep bruxism (SB) in an Egyptian population sample and to identify possible associations between SB, obstructive sleep apnea (OSA), and gastroesophageal reflux disease (GERD).

**Methods:**

This study was conducted on patients visiting dental clinics of three governmental institutions: the National Research Centre, Cairo University, and Ain-Shams University in Egypt. All dentulous patients aged 18-70 were included in the study; those with physical or mental disabilities or genetic disorders were excluded. Validated questionnaires and clinical dental examinations were used to diagnose SB, OSA, and GERD. Statistical analysis was performed to study possible associations. The significance level was set at *p* ≤ 0.05.

**Results:**

Nine hundred ninety-one subjects participated in this study. The prevalence of SB was 13.0%. The mean and standard deviation (SD) of the OSA score in sleep bruxers were 1.48 (1.55), as opposed to 1.35 (1.5) for non-sleep bruxers, showing no statistical significance with *p*= 0.388. The prevalence of GERD in the population was 22.7%; in sleep bruxers, 36.4% and in non-sleep bruxers, 20.7%, showing high statistical significance. The mean GERD score for sleep bruxers was 7.24 (2.36), as opposed to 6.69 (1.79) for non-sleep bruxers, also showing statistical significance with *p* = 0.002.

**Conclusions:**

The Egyptian population has a high prevalence of SB (13.0%). An association between SB and GERD was manifested. However, no relation between SB and OSA was found.

**KEYWORDS**

Sleep bruxism, OSA, GERD, sleep disorders, Egypt

**Authors details:**

-**Asmaa N. Elboraey**

**Email**: asmaanabil10@yahoo.com

**ORCID**: 0000-0002-1507-8957

Associate Professor, Fixed and Removable Prosthodontic Department, Oral and Dental Research Institute, National Research Centre, Cairo, Egypt.

**-Amani R. Moussa**

**Email**: amani.moussa66@yahoo.com

**ORCID: 0000-0003-1322-1008**

Professor, Fixed and Removable Prosthodontic Department, Oral and Dental Research Institute, National Research Centre, Cairo, Egypt.

**-Eman Mostafa**

**Email:** eman1mostafa@yahoo.com

**ORCID: 0000-0002-8605-4469**

Professor, Fixed and Removable Prosthodontic Department, Oral and Dental Research Institute, National Research Centre, Cairo, Egypt.

**-Menatallah Mohamed Elhotieby**

**Email**: [menat2005@gmail.com](mailto:menat2005@gmail.com)

**ORCID:** 0000-0003-3698-3259

Researcher, Fixed and Removable Prosthodontic Department, Oral and Dental Research Institute, National Research Centre, Cairo Egypt.

**Ahmed Gharib**

**Email:** [ahmedgharib@hotmail.com](mailto:ahmedgharib@hotmail.com)

ORCID: 0000-0003-4503-7715

Department of Internal Medicine, Medical Research and Clinical Studies Institute, National Research Centre

**Enas Sabry**

**Email:** Enassabry@dent.asu.edu.eg

Faculty of Dentistry, Ain-Shams University

**Noha Adel**

Email: [noha.adel@dentistry.cu.edu.eg](mailto:noha.adel@dentistry.cu.edu.eg)

Faculty of Oral and Dental Medicine, Cairo University

**Ali Yasser**

Email: ali.yasser@dentistry.cu.edu.eg

Faculty of Oral and Dental Medicine, Cairo University

**Magda I. Ramzy:**

**Email**: [magdaramzy@hotmail.com](mailto:magdaramzy@hotmail.com)

**ORCID:** 0000-0003-1243-4052

Professor, Fixed and Removable Prosthodontic Department, Oral and Dental Research Institute, National Research Centre, Cairo, Egypt.

**Hadiel Zamzam**

Email: [hadielzamzam@hotmail.com](mailto:hadielzamzam@hotmail.com)

ORCID: 0000-0003-2172-3569

Researcher, Fixed and Removable Prosthodontic Department, Oral and Dental Research Institute, National Research Centre, Cairo Egypt

**DECLARATIONS:**

**ETHICS APPROVAL AND CONSENT TO PARTICIPATE**

The project was approved by the Medical Research Ethics Committee of the NRC (MREC ethical approval number: 19242). All participants signed an informed consent form for their approval.

**CONSENT FOR PUBLICATION**

Not applicable

**AVAILABILITY OF DATA AND MATERIALS**

The datasets used and analyzed during the current study are available from the corresponding author upon reasonable request.

**COMPETING INTERESTS**

The authors declare that they have no known conflicts of interest or personal benefits that could have appeared to influence this work or its results.

**FUNDING**

This study was part of a research project (ID number: 12060208) funded by the National Research Centre in Egypt (NRC).

**AUTHORS’ CONTRIBUTIONS**

HZ: study idea, data collection, data analysis, data interpretation, manuscript revision. MH: data collection, data analysis. AE: data collection, data analysis. EM: data collection, data analysis, manuscript drafting. AG: study idea, data interpretation, manuscript drafting. ES: data collection. NA: data collection. AY: data collection. MR: study idea, data interpretation. AR: study idea, data collection, data analysis, data interpretation, manuscript revision.

**ACKNOWLEDGMENTS**

Funding for the whole research project (ID number: 12060208) was received from the National Research Centre in Egypt. For further information, please get in touch with Dr. Zamzam at [hadielzamzam@hotmail.com](mailto:hadielzamzam@hotmail.com)
